# Reviewer acknowledgement

**DOI:** 10.1186/1742-2094-10-17

**Published:** 2013-02-26

**Authors:** Sue T Griffin, Robert E Mrak

**Affiliations:** 1 University of Arkansas for Medical Sciences, Department of Geriatrics, 629 Jack Stephens Dr., AR 72205, Little Rock, USA; 2 Department of Pathology, University of Toledo Health Sciences Campus, 300 Arlington Ave, MS #1090, OH 43614-2598, Toledo, USA

## Abstract

**Contributing reviewers:**

The editors of Journal of Neuroinflammation would like to thank all the reviewers who have contributed to the journal in Volume 9 (2012).

## 

Smriti Agrawal

Canada

Masaaki Akagi

Japan

Silvia Alboni

Italy

Hakan Aldskogius

Sweden

Osborne Almeida

Germany

Oscar Alzate

United States of America

Fumimasa Amaya

Japan

Thomas Anderson

United Kingdom

Christa Anderson

United States of America

Anuska Andjelkovic Zochowska

United States of America

Katrin Andreasson

United States of America

Doychin Nikolov Angelov

Germany

Jack Antel

Canada

Katia Aquilano

Italy

Nathalie Arbour

Canada

Konstantinos Arfanakis

United States of America

Paul Ashwood

United States of America

Muhammad Aslam

Germany

Julie Atkin

Australia

Adam Bachstetter

United States of America

Emilio Badoer

Australia

Jong-Sup Bae

Korea, South

Michael Bailey

United States of America

Carmela Balistreri

Italy

William Banks

United States of America

Lisa Banner

United States of America

Steven Barger

United States of America

Roger Barker

United Kingdom

Anirban Basu

India

Sebastian Bauer

Germany

Ian Beales

United Kingdom

Kevin Becker

United States of America

Simon Beggs

Canada

Andreas Beineke

Germany

Jorge Benach

United States of America

Reina Bendayan

Canada

Caterina Bendotti

Italy

Rafael Benoliel

Israel

Anna-Lena Berg

Sweden

Vincent Beringue

France

Antonio Bertolotto

Italy

Cordian Beyer

Germany

Erwan Bezard

France

Knut Biber

Germany

Peter Biberthaler

Germany

Paula Bickford

United States of America

Helle Bielefeldt-Ohmann

Australia

Staci Bilbo

United States of America

Alexander Binshtok

Israel

Silvia Bisti

Italy

Marc Block

France

Detlev Boison

United States of America

Juan Bolanos

Spain

Diana Boraschi

Italy

Paola Bossu

Italy

William Bowers

United States of America

Alexander Brack

Germany

Don Branch

Canada

John Bright

United States of America

Andreas Bringmann

Germany

Raf Brouns

Belgium

Philip Brownjohn

New Zealand

Shilpa Buch

United States of America

Tom Burne

Australia

Ashley Bush

United States of America

Kimberly Byrnes

United States of America

Jean Lud Cadet

United States of America

Annachiara Cagnin

Italy

Sean Callanan

Ireland

Iain Campbell

Australia

Giuseppe Campo

Italy

Eduardo Candelario-Jalil

United States of America

Margherita Cantorna

United States of America

Valerie Capra

Italy

Cristiano Capurso

Italy

Steve Carlson

United States of America

Annabelle Caron

Canada

Monica Carson

United States of America

Calogero Caruso

Italy

Sonia Chalbot

United States of America

Andrew Chan

Germany

Sulie Chang

United States of America

Xiaochun Chen

China

Michael Chez

United States of America

Zhao Zhong Chong

United States of America

Julie Chowen

Spain

Marilyn Cipolla

United States of America

Fredrik Clausen

Sweden

Frederick Colbourne

Canada

Massimo Collino

Italy

Michael Collins

United States of America

Manuel Comabella

Spain

Colin Combs

United States of America

Deborah Commins

United States of America

Jim Connelly

United States of America

Bruno Conti

United States of America

Pio Conti

Italy

Angela Corona

United States of America

Fiona Costello

Canada

Rejean Couture

Canada

Charles Cox

United States of America

Colm Cunningham

Ireland

Imad Damaj

United States of America

Robert Dantzer

United States of America

Kunjan Dave

United States of America

Samuel David

Canada

George Davis

United States of America

Jeannette Davies

United States of America

Randall Davis

United States of America

Thomas Davis

United States of America

Maria Grazia De Simoni

Italy

Helga de Vries

Netherlands

Terrence Deak

United States of America

Mark DeCoster

United States of America

Isabelle Decosterd

Switzerland

Bernard Degryse

Ireland

Faramarz Dehghani

Germany

Florian Deisenhammer

Austria

Maria Delivoria-Papadopoulos

United States of America

Ronald Deumens

Belgium

A Courtney DeVries

United States of America

George DeVries

United States of America

Krishnan Dhandapani

United States of America

Simone Di Giovanni

Germany

Gilbert Di Paolo

United States of America

Miguel Diaz-Hernandez

Spain

Dara Dickstein

United States of America

Dalton Dietrich

United States of America

Jacopo C. DiFrancesco

Italy

Marcel Dihne

Germany

Ted Dinan

Ireland

Charles Dinarello

United States of America

Bonnie Dittel

United States of America

Axinia Doering

Canada

Rosario Donato

Italy

Katerina Dorovini-Zis

Canada

Patrick Dougherty

United States of America

Robert Doyle

United States of America

Alexander Dressel

Germany

Peter Drummond

Australia

Steven Dudek

United States of America

Lars Edvinsson

Sweden

Florian Eichler

United States of America

Piet Eikelenboom

Netherlands

James Eisenach

United States of America

Joseph El Khoury

United States of America

Brian Eliceiri

United States of America

Britta Engelhardt

Switzerland

Zelig Eshhar

Italy

Darryl Eyles

Australia

Paolo Fabene

Italy

Klaus Fassbender

Germany

S Hossein Fatemi

United States of America

Alexander Faussner

Germany

Pedro Michael Faustmann

Germany

Maurizio Federico

Italy

Gerald Feldman

United States of America

Markus Fendt

Germany

Maria Figueiredo-Pereira

United States of America

Andrea Flex

Italy

Tullio Florio

Italy

Candace Floyd

United States of America

Christian Foerch

Germany

Joseph Frank

United States of America

Matthew Frank

United States of America

Sally Frautschy

United States of America

Willard Freeman

United States of America

Dan Frenkel

Israel

Gregory Freund

United States of America

Yvonne Freund-Levi

Sweden

Kaj Fried

Sweden

Elliot Frohman

United States of America

Shin-ichiro Fujii

Japan

Ken-ichiro Fukuchi

United States of America

Kohji Fukunaga

Japan

Lorenzo Fumagalli

Italy

Roberto Furlan

Italy

Luis Miguel Garcia-Segura

Spain

GA Garden

United States of America

Natalie Gardiner

United Kingdom

Peter Gaskill

United States of America

Achim Gass

Germany

Wagner Gattaz

Brazil

Jonathan Geiger

United States of America

Herbert Geller

United States of America

John Gensel

United States of America

Ruediger Gerstberger

Germany

Changiz Geula

United States of America

Anuja Ghorpade

United States of America

Simantini Ghosh

United States of America

Othman Ghribi

United States of America

De Sarro Giovambattista

Italy

Candece Gladson

United States of America

Jonathan Godbout

United States of America

Ralf Gold

Germany

James Goldman

United States of America

Elise Gomez-Sanchez

United States of America

Cheng-Xin Gong

United States of America

Oscar Gonzalez-Perez

Mexico

Dayan Goodenowe

Canada

Marcelo Gottschalk

Canada

Manuel Graeber

Australia

Paula Grammas

United States of America

Francesc Graus

Spain

Kim Green

United States of America

Nigel Greig

United States of America

Pierre Gressens

France

Wolfgang Grisold

Austria

Claudia Grothe

Germany

Virginia Guarani-Pereira

United States of America

Carmen Guaza

Spain

Balazs Gulyas

Sweden

Raphael Guzman

Switzerland

Randi Hagerman

United States of America

Nils Hailer

Sweden

Marc Halterman

United States of America

Michael Hamblin

United States of America

Inn-Oc Han

Korea, South

Ryosuke Hanaya

Japan

Uwe-Karsten Hanisch

Germany

James Haorah

United States of America

Akira Hara

Japan

G Jean Harry

United States of America

Adam Hartman

United States of America

Hans-Peter Hartung

Germany

Johnny He

United States of America

Yun He

United States of America

Michael Hecker

Germany

Cobi Heijnen

Netherlands

Seppo Helisalmi

Finland

Michael Heneka

Germany

Kenneth Hensley

United States of America

Miles Herkenham

United States of America

Emmanuel Hermans

Belgium

Ellen Hessel

Netherlands

Sandra Hewett

United States of America

David Hinkle

United States of America

Toru Hiraga

Japan

Yoshitaka Hirooka

Japan

Wen-Zhe Ho

United States of America

Karin Hochrainer

United States of America

Barry Hoffer

United States of America

Harald Hofstetter

Germany

Ahmet Hoke

United States of America

Clive Holmes

United Kingdom

Irma E. Holopainen

Finland

Jau-Shyong Hong

United States of America

Koji Hosaka

United States of America

Charles Howe

United States of America

Ching-Liang Hsieh

Taiwan

Huijuan Hu

United States of America

Stephane Hunot

France

Shahid Husain

United States of America

Mark Hutchinson

Australia

Chrysanthy Ikonomidou

United States of America

David Irani

United States of America

Hiroshi Ishii

Japan

Shingo Ito

Canada

Takayuki Itoh

United States of America

Kerstin Iverfeldt

Sweden

Koichi Iwata

Japan

Wilhelm Jahnen-Dechent

Germany

Sebastian Jander

Germany

Gary Jarvis

United States of America

Bhavaani Jayaram

United States of America

Glen Jeffery

United Kingdom

Ru-Rong Ji

United States of America

Glen Jickling

United States of America

Gareth John

United States of America

Erik Johnson

United States of America

Micahel VanDoren Johnston

United States of America

David Jones

United States of America

Barbaar Kaltschmidt

Germany

Lohitash Karumbaiah

United States of America

Guna Karupiah

Australia

Hiroshi Katsuki

Japan

Dan Kaufman

United States of America

Marcus Kaul

United States of America

Charanjit Kaur

Singapore

Srini Kaveri

France

Robert Keane

Afghanistan

Brian Kelley

United States of America

Oliver Kempski

Germany

Tim Kern

United States of America

Mushfiquddin Khan

United States of America

Mahmoud Kiaei

United States of America

Aize Kijlstra

Netherlands

Yoon-Seong Kim

United States of America

Hideo Kimura

Japan

Jonathan Kipnis

United States of America

Joachim Kirsch

Germany

Robyn Klein

United States of America

Lars Klimaschewski

Austria

Raoul Peter Kloppenborg

Netherlands

Luisa Klotz

Germany

Matthias Klugmann

Australia

Uwe Koedel

Germany

Schuichi Koizumi

Japan

Shin-ichi Konno

Japan

Jan Pieter Konsman

France

Tomasz Kordula

United States of America

Thomas Korn

Germany

Czeslawa Kowal

United States of America

Michaela Kress

Austria

Gurumoorthy Krishnamoorthy

Germany

Jasna Kriz

Canada

Paul Kubes

Canada

Adarsh Kumar

United States of America

Ashok Kumar

United States of America

Wolfram S Kunz

Germany

Jose L Labandeira-Garcia

Spain

Steve Lacroix

Canada

Monique Lafon

France

Debomoy Lahiri

United States of America

Bruce Lamb

United States of America

Thomas Lane

United States of America

Thomas Langmann

Germany

Max Larsson

Sweden

Corinne Lasmezas

United States of America

Hans Lassmann

Austria

Justin Lathia

United States of America

Matthew LaVoie

United States of America

Hyoung-gon Lee

United States of America

Sunhee Lee

United States of America

Cynthia Lemere

United States of America

Alan Leviton

United States of America

Joanna Lewin-Kowalik

Poland

Juan C Leza

Spain

Jian Liang

United States of America

Federico Licastro

Italy

Stefan Liebau

Germany

Jonathan Lifshitz

United States of America

Raija LP Lindberg

Switzerland

Ralf Linker

Germany

Robert Lisak

United States of America

Fei Liu

United States of America

David Loane

United States of America

David Loeffler

United States of America

James Lokensgard

United States of America

Stefan Lorkowski

Germany

Binfeng Lu

United States of America

Ru-Band Lu

Taiwan

Lih-Fen Lue

United States of America

Giamal Luheshi

Canada

Thomas Lukas

United States of America

Hans U Lutz

Switzerland

Marina Lynch

Ireland

Andrew Maclean

United States of America

Mathias Maeurer

Germany

Sanjay Maggirwar

United States of America

Amir Hadi Maghzi

Iran

Kathleen Maguire-Zeiss

United States of America

Carina Mallard

Sweden

Yogesh Mariappan

United States of America

Fernanda Marques

Portugal

Ian Marriott

United States of America

Trevor Marshall

United States of America

Saarma Mart

Finland

Monica Marta

United Kingdom

Maria Marti

Spain

Terina Martinez

United States of America

Jose Martinez-Gonzalez

Spain

Rudolf Martini

Germany

Paul Massa

United States of America

Lourdes Massieu

Mexico

Toshio Matsuda

Japan

Anne Kathrin Mausberg

Germany

Frederick Maxfield

United States of America

Margot Mayer-Proschel

United States of America

James (Pat) McAllister

United States of America

Thomas McCown

United States of America

Louise McCullough

United States of America

Steve McGaraughty

United States of America

Edgar Meinl

Germany

Til Menge

Germany

Christine Metz

United States of America

Sven Meuth

Germany

Gerd Meyer zu Hörste

Germany

Vjekoslav Miletic

United States of America

Richard Miller

United States of America

Richard Milner

United States of America

Luisa Minghetti

Italy

Thomas Moeller

United States of America

Brit Mollenhauer

Germany

Juliet Moncaster

United States of America

Nancy Monson

United States of America

Craig Moore

Canada

Maria Cristina Morganti-Kossmann

Australia

Maria Angeles Moro

Spain

Gehan Ahmed Mostafa

Egypt

Monique Mulder

Netherlands

Victoriano Mulero

Spain

Marcus Müller

Germany

Anjana Munshi

India

Makoto Murakami

Japan

Eric Murphy

United States of America

Greer Murphy

United States of America

Erik Musiek

United States of America

Manohar Mutnal

United States of America

Aye Mu Myint

Germany

Aryan Namboodiri

United States of America

Mahesh Narayan

United States of America

Roland Nau

Gibraltar

Lucia Negri

Italy

Randy Nelson

United States of America

Elizabeth Neufeld

United States of America

Robert Nibbs

United Kingdom

Kimberly Nixon

United States of America

Linda Noble

United States of America

Tadashi Nomura

Japan

Michael Norenberg

United States of America

Daniela Novick

Israel

M. Kerry O'Banion

United States of America

Fumiya Obata

Japan

Otilia Obreja

Germany

James O’Callaghan

United States of America

Mauro Oddo

Switzerland

Salvatore Oddo

United States of America

Halina Offner

United States of America

John Olschowka

United States of America

Bob Olsson

Sweden

Sandra Olsson

Sweden

Roald Omdal

Norway

Lisa Opanashuk

United States of America

Mark Opp

United States of America

Celia Oreja-Guevara

Spain

Suidong Ouyang

United States of America

Trevor Owens

Denmark

Kalipada Pahan

United States of America

Alan Palmer

United Kingdom

Pyong Woo Park

United States of America

Lucilla Parnetti

Japan

Vladimir Parpura

United States of America

Manisha Patel

United States of America

Jogi Pattisapu

United States of America

Surojit Paul

United States of America

Ragnhild Paulsen

Norway

Keith Pennypacker

United States of America

Guey Chuen Perng

United States of America

Yuri Persidsky

United States of America

Klaus G Petry

France

Mario Philipp

United States of America

Fredrik Piehl

Sweden

Fernando Pitossi

Argentina

Anna Planas

Spain

David Pleasure

United States of America

Stefano Pluchino

United Kingdom

Jennifer Pocock

United Kingdom

Michel Pohl

France

Roberto Pola

United States of America

Nunzio Pomara

United States of America

Forbes Porter

United States of America

Heidrun Potschka

Germany

Christopher Power

Canada

Sandeep Prabhu

United States of America

Mark Prendergast

United States of America

Lynn Pulliam

United States of America

Lintao Qu

United States of America

Anthony Quinn

United States of America

Marko Radic

United States of America

Geoffrey Raisman

United Kingdom

Gennadij Raivich

United Kingdom

Mohan Raizada

United States of America

Dayanidhi Raman

United States of America

Matt Ramer

Canada

Markus Reindl

Austria

Norman Relkin

United States of America

Kiarash Riazi

Canada

Joaquim Ribeiro

Portugal

Daniel Rittirsch

Switzerland

Marie-Françoise Ritz

Switzerland

Maha Rizk

Egypt

Prema Robinson

United States of America

Jean-Christophe Rochet

United States of America

Joseph Rogers

United States of America

Julian Romero

Spain

Gary Rosenberg

United States of America

Carole Rovere

France

Walter Royal

United States of America

Marc Ruitenberg

Australia

Christoph Rummel

Germany

John Russell

United States of America

Ricardo Saban

United States of America

Jacqueline Sagen

United States of America

Samina Salim

United States of America

Frederic Saltel

France

Marta San Luciano

United States of America

Enrique Sanchez-Lemus

United States of America

Lauren Sansing

United States of America

Devanand Sarkar

United States of America

Josep Saura

Spain

Bassel E Sawaya

United States of America

Morris Scantlebury

Canada

Scott Schachtele

United States of America

Sven Schippling

Switzerland

Dirk Schlüter

Germany

Rachel Schrier

United States of America

Fatima Sehba

United States of America

Johann Sellner

Germany

Bridgette Semple

United States of America

Satoru Senju

Japan

Florian Sennlaub

France

Thomas Seyfried

United States of America

Lee Shapiro

United States of America

Becham Sharma

India

Chan Shin

Korea, South

Yasushi Shintani

Japan

Seiji Shioda

Japan

Yehuda Shoenfeld

Israel

Veronica Shubayev

United States of America

Sandy Shultz

Australia

R. Douglas Shytle

United States of America

Peter Silverstein

United States of America

James Simpkins

United States of America

Ian Simpson

United States of America

Rajendra Singh

India

Ajay Singh

United States of America

Inderjit Singh

United States of America

Mahendra Singh

India

Richard Smeyne

United States of America

A Gordon Smith

United States of America

Alicia Smith

United States of America

Carolyn Beebe Smith

United States of America

Craig Smith

United Kingdom

Anja Smits

Sweden

Jessica Snowden

United States of America

Tomás Sobrino

Spain

Michael V. Sofroniew

United States of America

Farida Sohrabji

United States of America

Carme Sola

Spain

Claudia Sommer

Germany

Maria Angela Sortino

Italy

Philip Stahel

United States of America

Martin Stangel

Germany

Johann Steiner

Germany

Christian Steinhäuser

Germany

Michael Stewart

United Kingdom

Stephen Stohlman

United States of America

Guido Stoll

Germany

Barbara Stonestreet

United States of America

Wolfgang J. Streit

United States of America

Olaf Stuve

United States of America

Hyeon-Sook Suh

United States of America

Kyoungho Suk

Korea, South

Patrick Sullivan

United States of America

Grace Sun

United States of America

Katsuaki Suzuki

Japan

Hidenori Suzuki

Japan

Akio Suzumura

Japan

Peter Syapin

United States of America

Arantxa Tabernero

Spain

Björn Tackenberg

Germany

Hisaaki Takahashi

Japan

Stefano Tamburin

Italy

Jun Tan

Vanuatu

Masako Taniike

Japan

Kunikazu Tanji

Japan

Malu Tansey

United States of America

Dennis Taub

United States of America

Peter Teismann

United Kingdom

Andrea Tenner

United States of America

David Thomas

United States of America

Kim Tieu

United States of America

Cynthia Tifft

United States of America

Stephanie Tjen-A-Looi

United States of America

Michal Toborek

United States of America

Mary Tolcos

Australia

Hiroyuki Tomiyama

Japan

Rosa J Torres

Spain

Isabelle Touitou

France

Terrence Town

United States of America

Andreas Traweger

Austria

Corinna Trebst

Germany

Flavia Trettel

Italy

Ching Piao Tsai

Taiwan

Antonino Tuttolomondo

Italy

Monica Uddin

United States of America

Oliver Ullrich

Switzerland

Peter Vajkoczy

Germany

Walter van den Bergh

Netherlands

Erwin van Vliet

Netherlands

Jonathan Vappou

France

David Vaudry

France

Libor Velisek

United States of America

Roland Veltkamp

Germany

Jose L. Venero

Spain

Arun Venkatesan

United States of America

Alexei Verkhratsky

United Kingdom

Saguna Verma

United States of America

Timothy Verstynen

United States of America

Annamaria Vezzani

Italy

Robert Vink

Australia

Barbara Viviani

Italy

H.-Christian von Büdingen

United States of America

Mark Wainwright

United States of America

Abdelilah Wakkach

France

Haibo Wang

United States of America

Ping Wang

United States of America

Yun Wang

United States of America

Zaijie Wang

United States of America

Zunyi Wang

United States of America

Clemens Warnke

Germany

Linda Watkins

United States of America

Jyoti Watters

United States of America

Lindell Weaver

United States of America

Joerg Weber

Germany

Martin S. Weber

Germany

Andrea Weinstein

United States of America

Janis Weis

United States of America

Wolfgang Weninger

Australia

Gary Wenk

United States of America

Martin Wessendorf

United States of America

Fletcher White

United States of America

Paul Whiteaker

United States of America

Marlene Wiart

France

Heinz Wiendl

Germany

Donna M Wilcock

United States of America

Frederick Williams

United States of America

H. Richard Winn

United States of America

Wai Wong

United States of America

John Wood

United Kingdom

Nicola Woodroofe

United Kingdom

Barbara Woodside

Canada

Hans Worthmann

Germany

Douglas Wright

United States of America

Jens Wuerfel

Germany

Shirley ShiDu Yan

United States of America

Qingwu Yang

China

Chuen-Mao Yang

Taiwan

Midori Yenari

United States of America

V Wee Yong

Canada

Byong Chul Yoo

Korea, South

Thomas Yorio

United States of America

Zengqiang Yuan

China

Giampietro Zanette

Italy

Renata Zanon

Brazil

Christoph Michael Zehendner

Germany

Deliang Zhang

United States of America

Li Zhang

United States of America

Haitao Zhu

United States of America

Xiaoping Zhu

United States of America

Xiongwei Zhu

United States of America

Jenna Ziebell

United States of America

Frauke Zipp

Germany

Irving Zucker

United States of America

